# A chemical genetic screen uncovers a small molecule enhancer of the *N*-acylethanolamine degrading enzyme, fatty acid amide hydrolase, in Arabidopsis

**DOI:** 10.1038/srep41121

**Published:** 2017-01-23

**Authors:** Bibi Rafeiza Khan, Lionel Faure, Kent D. Chapman, Elison B. Blancaflor

**Affiliations:** 1 Plant Biology Division, The Samuel Roberts Noble Foundation Inc., Ardmore, OK, 73401, USA; 2 Center for Plant Lipid Research, Department of Biological Sciences, University of North Texas, Denton, TX 76203, USA; 3 Biology Department, Texas Woman’s University, Denton, TX 76204, USA

## Abstract

*N*-Acylethanolamines (NAEs) are a group of fatty acid amides that play signaling roles in diverse physiological processes in eukaryotes. Fatty acid amide hydrolase (FAAH) degrades NAE into ethanolamine and free fatty acid to terminate its signaling function. In animals, chemical inhibitors of FAAH have been used for therapeutic treatment of pain and as tools to probe deeper into biochemical properties of FAAH. In a chemical genetic screen for small molecules that dampened the inhibitory effect of *N*-lauroylethanolamine (NAE 12:0) on *Arabidopsis thaliana* seedling growth, we identified 6-(2-methoxyphenyl)-1,3-dimethyl-5-phenyl-1H-pyrrolo[3,4-d]pyrimidine-2,4(3 H,6 H)-dione (or MDPD). MDPD alleviated the growth inhibitory effects of NAE 12:0, in part by enhancing the enzymatic activity of *Arabidopsis* FAAH (AtFAAH). *In vitro,* biochemical assays showed that MDPD enhanced the apparent V_max_ of AtFAAH but did not alter the affinity of AtFAAH for its NAE substrates. Structural analogs of MDPD did not affect AtFAAH activity or dampen the inhibitory effect of NAE 12:0 on seedling growth indicating that MDPD is a specific synthetic chemical activator of AtFAAH. Collectively, our study demonstrates the feasibility of using an unbiased chemical genetic approach to identify new pharmacological tools for manipulating FAAH- and NAE-mediated physiological processes in plants.

*N*-Acylethanolamines (NAEs) represent a group of fatty acid amides conserved among eukaryotes^1^,^2^. NAEs are part of the endocannabinoid signaling pathway in vertebrates where they modulate a plethora of behavioral and physiological processes such as appetite, mood, cardiovascular function, sleep and reproduction^3^,^4^. One of the most intensively studied NAE species is *N*-arachidonoylethanolamine (NAE 20:4), or anandamide. Anandamide binds to the cannabinoid receptor 1 (CB1), in the brain to aid in perception of pain and stimulation of pleasure. NAE signaling in mammals is terminated by the fatty acid amide hydrolase (FAAH) enzyme to yield ethanolamine and the corresponding free fatty acid^5^,^6^,^7^. Several lines of evidence support the role of FAAH in regulating endogenous NAE levels and consequently, NAE-mediated physiological processes. The most compelling are studies with mice where it was shown that mutations in the *FAAH* gene, led to the accumulation of endogenous fatty acid amides in the central nervous system, and the reduction of the animal’s sensitivity to anandamide-dependent neurological processes such as pain sensation^8^,^9^.

Recent work in *Arabidopsis thaliana* has shown that NAEs play a role in several biological pathways including processes associated with early seedling development, flowering time, and responses to bacterial pathogens^10^,^11^,^12^. There is also evidence that NAEs could exert their signaling function in plants via cross-talk with hormones like abscisic acid (ABA)^13^,^14^ or salicylic acid^11^. Perhaps the most significant advance with regard to uncovering NAE’s role in plants is the discovery of plant genes encoding proteins with strong similarity to the amidase signature domain of mammalian FAAH^15^,^16^. Functional analyses of one *Arabidopsis FAAH (AtFAAH*) encoded by the gene *At5g64440* led to modified responses of seedlings to exogenous NAE. For instance, seedlings of transfer (T)-DNA knockouts to *AtFAAH (Atfaah*) were more severely inhibited by *N*-lauroylethanolamine (NAE 12:0) compared to wild type, whereas *AtFAAH* overexpressor seedlings were more resistant^17^. Furthermore, the finding that *Atfaah* had elevated, while *AtFAAH* overexpressors had lower endogenous NAEs, respectively, indicate that AtFAAH is an important enzyme involved in NAE hydrolysis^12^,^17^.

Pharmacological studies using chemical inhibitors to mammalian FAAH have had major therapeutic implications for the treatment of pain and various neuropsychiatric disorders. Early examples of FAAH chemical inhibitors include sulfonyl fluorides^18^, trifluoromethyl ketones^19^, fluorophosphonates^18^, and most notably, carbamates (URB532 and URB597)^20^. As expected from a FAAH inhibitor, rodents treated with carbamate inhibitors accumulated endogenous anandamide, and other NAE species in the brain resulting in anxiolytic and analgesic responses. More recently, FAAH inhibitors like OL-135, which reduces nociceptive response, and PF-3845, a highly selective FAAH inhibitor with a longer duration of action, significantly dampened inflammatory pain^21^,^22^,^23^,^24^,^25^. While some active-site directed inhibitors of mammalian FAAH also will inhibit AtFAAH activity, to date, chemicals that specifically modify plant FAAH enzymatic activity have yet to be identified.

In this paper, we present results on a small molecule that enhances the enzymatic activity of AtFAAH. This molecule, which we called MDPD, was isolated from a chemical genetic screen of a library of 10,000 membrane permeable synthetic compounds to test for interference with the inhibitory effects of NAE 12:0 on *Arabidopsis* seedling growth. It was found that the ability of MDPD to dampen the growth inhibitory effects of NAE 12:0 on seedling growth can be explained in part by its enhancement of AtFAAH activity. To our knowledge, MDPD is the first synthetic molecule that stimulates the activity of a FAAH protein and therefore provides a novel tool to probe deeper into the biochemical properties and functions of plant FAAH enzymes.

## Results

### MDPD attenuates the inhibitory effects of NAE 12:0 on *Arabidopsis* seedling growth

**Figure 1:**
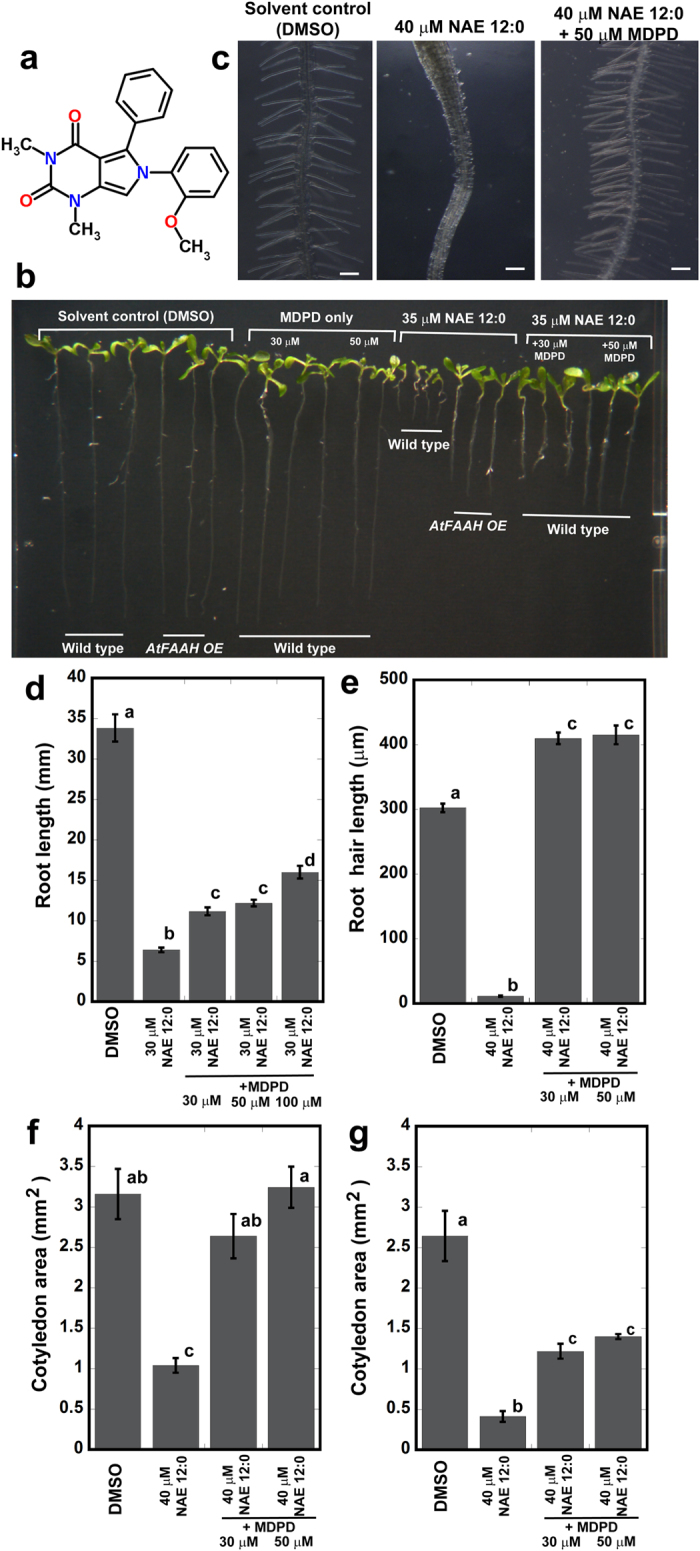
Effects of MDPD on NAE 12:0-induced seedling growth inhibition. (**a**) Structure of 6-(2-methoxyphenyl)-1,3-dimethyl-5-phenyl-1H-pyrrolo[3,4-d]pyrimidine-2,4(3 H,6 H)-dione (MDPD). (**b**) Wild type and *AtFAAH* overexpressors *(AtFAAH OE)* seedlings grown for 10 days with or without NAE 12:0 and MDPD. Note that wild-type seedlings on NAE 12:0 and MDPD mirror the growth of *AtFAAH OE* on NAE 12:0 alone. (**c**) NAE 12:0 inhibits root hair formation in wild-type seedlings but this is reversed in seedlings treated with both NAE 12:0 and MDPD. (**d**) Root length of wild-type seedlings on 30 μM NAE 12:0 alone or 30 μM NAE 12:0 plus 30 μM, 50 μM or 100 μM MDPD. Note that increasing MDPD concentrations progressively attenuates NAE 12:0-induced inhibition of root elongation. (**e**) Root hair length in MDPD and NAE 12:0 is significantly longer than root hair length in solvent control (DMSO) and reversed NAE 12:0-induced root hair growth inhibition. (**f,g**) Cotyledon area of seedlings on media supplemented with 30 μM or 40 μM NAE 12:0 and different concentrations of MDPD. Adding MDPD to the growth medium lessens the growth inhibitory effects of NAE 12:0 on cotyledon size. Error bars represent the standard error of the means (*n* ≥ 16 for cotyledon area; *n* ≥ 120 for primary root and root hair length. Means with different letters are significantly different (P < 0.005; Tukey’s test).

Exogenous NAE 12:0 inhibits *Arabidopsis* seedling growth^10^. Therefore, we used the growth inhibitory effects of NAE 12:0 as a basis for chemical screening of compounds that could impact NAE- mediated biological processes by germinating wild-type (Col-0) seeds in 96-well plates containing 50 μM NAE 12:0 together with one synthetic, small molecule at a final concentration of 100 μM and examined seedlings after 5 days. Ten small molecules that interfered with the growth inhibitory effects of NAE 12:0 were identified from this screen. After more stringent growth assays, we focused on the characterization of the small molecule, 6-(2-methoxyphenyl)-1, 3-dimethyl-5-phenyl-1H-pyrrolo [3,4-d]pyrimidine-2,4(3 H,6 H)-dione, or MDPD and its impact on NAE 12:0-mediated seedling growth inhibition (Fig. 1a; Figure S1). We found that MDPD was able to attenuate all aspects of the inhibitory effect of NAE 12:0 on seedling development. For example, at 30 μM NAE 12:0, primary roots of wild type seedlings were significantly reduced compared with seedlings grown in solvent control solutions, consistent with previous studies^10^. However, primary root length was longer for seedlings grown in both NAE 12:0 and MDPD compared to those in NAE 12:0 alone. The degree of primary root growth rescue was more pronounced with increasing concentrations of MDPD (Fig. 1b,d). Whereas MDPD only partially dampened the inhibitory effect of NAE 12:0 on primary root length, it completely reversed NAE 12:0-induced root hair defects (Fig. 1c,e). MDPD not only reversed the negative impact of NAE 12:0 on root hair elongation, but also significantly enhanced root hair growth compared to wild-type seedlings in solvent control solutions (Fig 1c,e).

Cotyledon expansion is also strongly inhibited by NAE 12:0^17^, therefore, we measured cotyledon area of wild-type seedlings treated with NAE 12:0 and MDPD. Seedlings treated with 30 μM and 40 μM NAE 12:0 only, showed a marked reduction in cotyledon expansion^17^. However, this NAE 12:0-induced inhibition of cotyledon expansion was prevented when MDPD was added to the growth media. Similar to observations made with root hairs, cotyledon expansion was restored to levels similar to control seedlings (Fig. 1b,f). The ability of MDPD to rescue the inhibitory effect of NAE 12:0 on cotyledon expansion was more dramatic at lower NAE 12:0 concentrations (e.g. 30 μM compared to 40 μM; Fig. 1f,g). MDPD also was able to dampen the inhibitory effect of NAE 12:0 on seedling growth when it was applied after NAE 12:0 had already initiated growth inhibition. For instance, when 30 μM of MDPD was added to seedlings pretreated with NAE 12:0 and seedlings were observed for another 3 days, seedlings treated with MDPD had more vigorous growth than seedlings exposed to solvent control solutions (Figure S2). In another set of experiments, seedlings were first pretreated with MDPD for 3 days and transplanted on NAE 12:0 supplemented plates. Under these conditions, MDPD pretreated seedlings transferred to NAE 12:0 were less inhibited compared to seedlings pretreated with solvent control solutions (Figure S3).

### The enzymatic activity of recombinant AtFAAH is enhanced by MDPD

**Figure 2:**
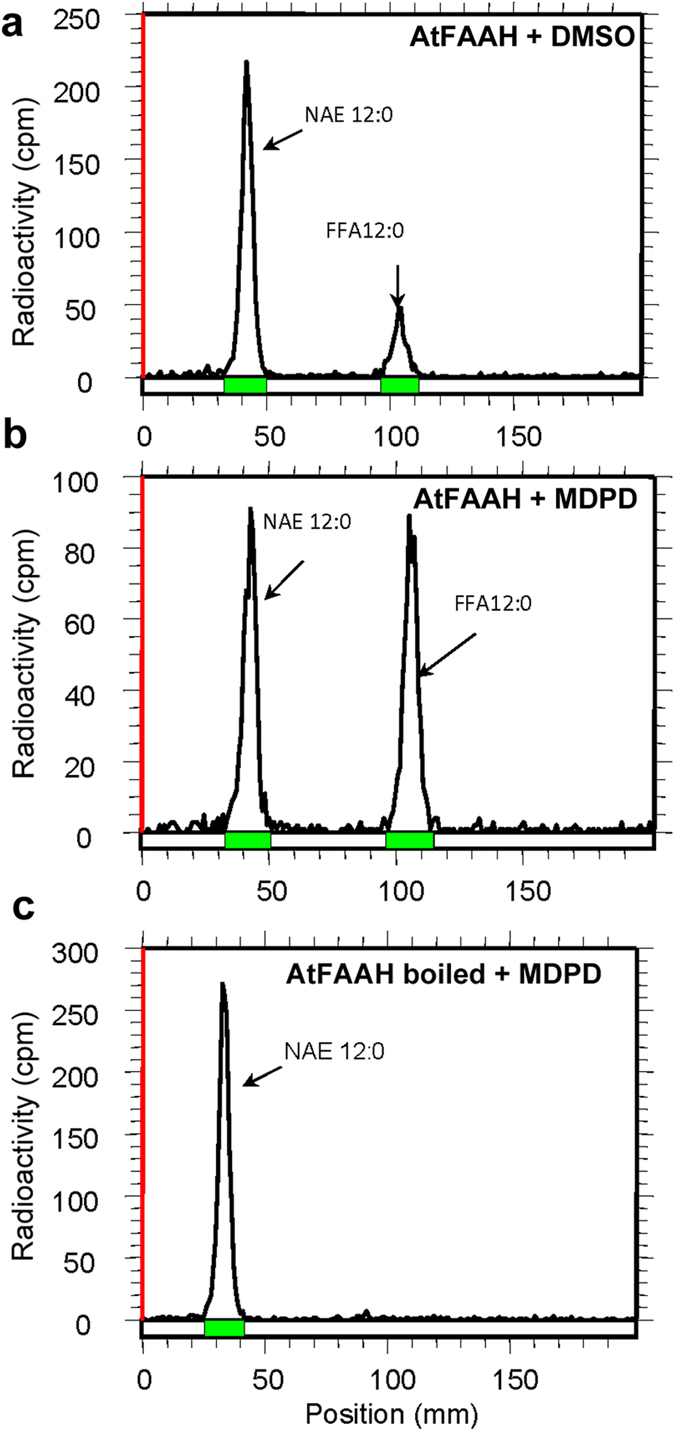
Representative radiochromatograms of amidohydrolase activity of recombinant AtFAAH to NAE 12:0. The reactions were initiated by the addition of 0.3 μg of purified AtFAAH with: (**a**) DMSO (solvent control); (**b**) 100 μM MDPD; and (**c**) 100 μM MDPD and boiled AtFAAH protein (negative control). The higher FFA peak in assays with MDPD (arrow in the peak to the right in (**b**) indicates enhanced enzymatic activity.

Because the reversal of seedling growth by MDPD was reminiscent of the more robust growth of *AtFAAH* overexpressors on exogenous NAE 12:0 (Fig. 1b)^17^, we hypothesized that AtFAAH might be a target of MDPD. To test this hypothesis, we conducted *in vitro* amidohydrolase assays with recombinant AtFAAH in the presence of MDPD using NAE 12:0 ([^14^-C]) as a substrate. The effect of MDPD on AtFAAH protein activity was determined by measuring the conversion of [^14^C] NAE 12:0 to [^14^C] free fatty acid (FFA). As shown by representative radiochromatograms in Fig. 2, we observed an increase in [^14^C]-FFA formation in the presence of MDPD compared to assays without MDPD (Fig. 2a,b). However, no conversion of [^14^C]-NAE 12:0 was detected in assays when inactive AtFAAH protein was used (Fig. 2c).

**Figure 3:**
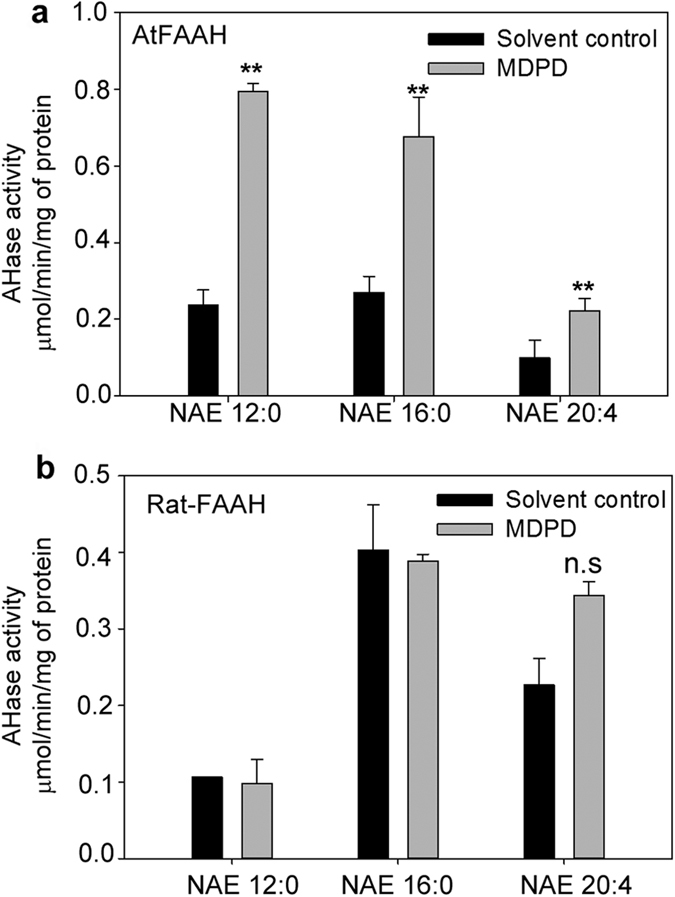
Amidohydrolase activity (AHase) with recombinant AtFAAH, Rat-FAAH, and different [1-^14^C]- NAEs. (**a**) In addition to NAE 12:0, AHase activity of AtFAAH in the presence of MDPD is enhanced using NAE 16:0 and NAE 20:4 as substrates. Data points represent means ± S.D. of triplicate assays. (**b**) Activity of recombinant rat-FAAH does not increase with MDPD addition. Data points represent means ± S.D. of triplicate assays. Student’s t-test (**indicates statistically significant differences, p < 0.05). Not significant (n.s.).

From the amount of [^14^C]-FFA formed for each assay, the activity of the enzyme (μmol/min/mg of protein) was determined. An increase by a factor of ~4 was calculated for AtFAAH activity in the presence of 100 μM MDPD and 100 μM of [^14^C]-NAE 12:0 (Fig. 3a). Our data suggest that AtFAAH is indeed a target of MDPD and it acts by enhancing the activity of AtFAAH for the NAE 12:0 substrate. AtFAAH enzymatic activity toward other NAE species was similarly enhanced by MDPD. For example, MDPD also enhanced AtFAAH activity for *N*-palmitoylethanolamine (NAE 16:0) and anandamide (NAE 20:4) in amidohydrolase assays. However, for NAE 20:4, which is not an endogenous NAE in plant tissues, AtFAAH activity was significantly, but not dramatically enhanced by MDPD (Fig. 3a). *In vitro* amidohydrolase assays with [^14^C]-NAE 12:0, - NAE 16:0, and – NAE 20:4 also were performed with the recombinant rat-FAAH enzyme with or without MDPD^26^. However, in our assay conditions, we did not detect any significant enhancement of rat-FAAH activity by MDPD toward NAE 12:0 and NAE 16:0. There was a slight but statistically insignificant increase in rat-FAAH activity toward NAE 20:4 in the presence of MDPD (Fig. 3b).

**Figure 4:**
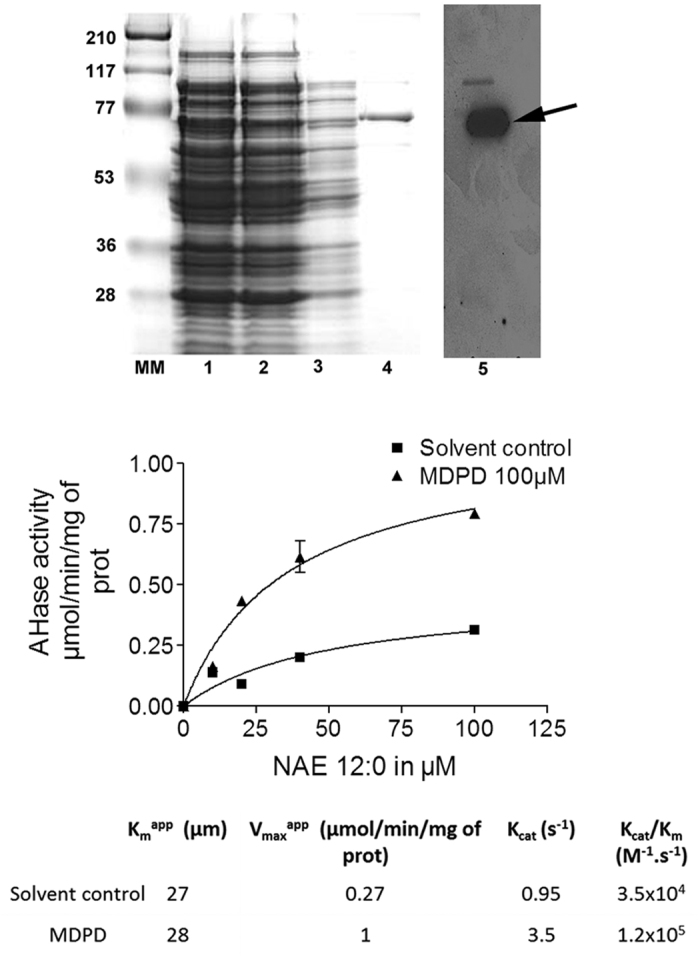
Coomassie-blue-stained SDS gel and Western blot analysis of AtFAAH probed with anti-HIS antibodies and kinetic characterization of recombinant AtFAAH with 100 μM MDPD. The epitope-tagged, recombinant AtFAAH protein used in the assays is predicted to be ~70 kDa (arrow). 1. crude protein extract; 2. supernatant fraction; 3. flow through; 4. eluted fraction (AtFAAH); 5, Western blot of eluted fraction; MM, Molecular marker. Initial velocities were plotted at increasing concentrations of [1-^14^C]-NAE 12:0 with and without MDPD. Reactions were initiated by the addition of AtFAAH. Catalytic parameters were calculated from these initial velocity data. Data points represent means ± S.D. of triplicate assays.

To gain more insight into how MDPD modulates AtFAAH, we determined the kinetic parameters of AtFAAH with and without MDPD. Consistent with previous studies, recombinant AtFAAH exhibited a typical Michaelis-Menten behavior toward the NAE 12:0 substrate (Fig. 4)^15^,^16^. In the presence of MDPD, the apparent V_max_ for AtFAAH was increased, but the apparent K_m_ (27–28 μM) of AtFAAH remained unchanged, indicating that MDPD enhanced the rate of hydrolysis, but not the substrate affinity of AtFAAH. Indeed, MDPD increased the catalytic efficiency of At FAAH by an estimated factor of ~3.4 (Fig. 4).

**Figure 5:**
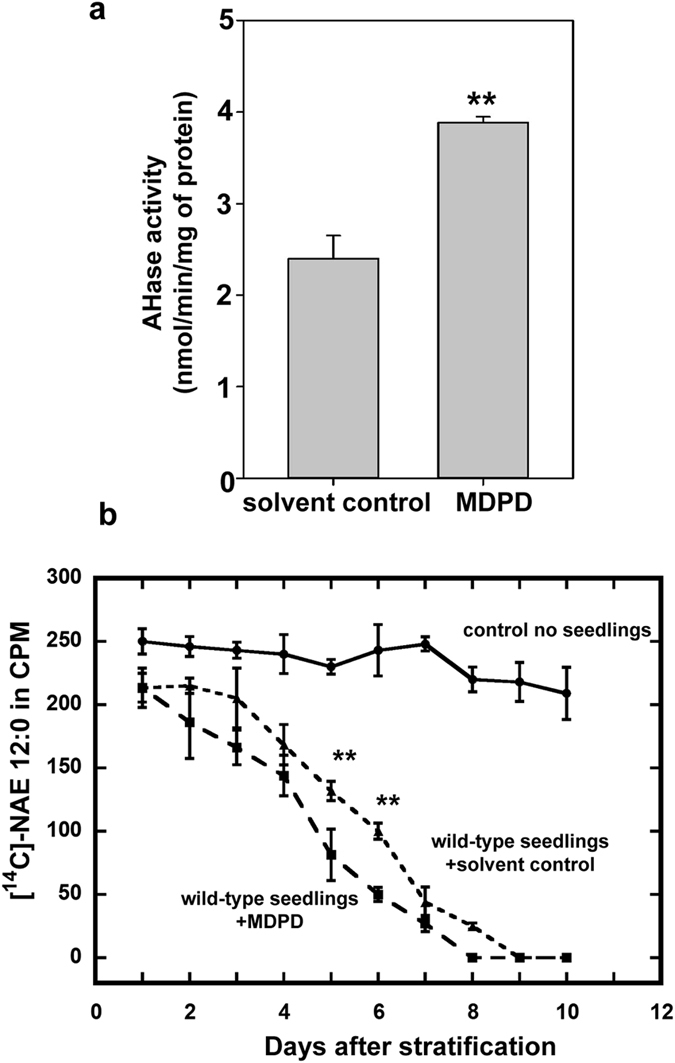
Amidohydrolase (AHase) assays on total seedling protein extracts and *in vivo* [1-^14^C]-NAE 12:0 depletion assays. (**a**) Increased AHase activity in crude protein extracts with MDPD. (**b**) [1-^14^C]-NAE 12:0 is depleted in liquid medium containing wild-type seedlings. Note that depletion of radiolabeled NAE 12:0 is faster in the presence of MDPD. Student’s t-test (**indicates statistically significant differences, p < 0.05).

Given that MDPD could reverse the inhibitory effects of NAE 12:0 on seedling growth, we next tested whether MDPD could also enhance endogenous amidohydrolase activity. We extracted total protein from 10 d old wild-type seedlings and conducted amidohydrolase assays on protein extracts. In agreement with assays of the recombinant AtFAAH protein, MDPD increased NAE hydrolytic activity in cell-free homogenates of seedlings (Fig. 5a). The ability of MDPD to enhance *in vivo* amidohydrolase activity was also apparent from experiments wherein the depletion of [1-^14^C]-NAE 12:0 was followed in liquid cultures containing wild-type *Arabidopsis* seedlings. Our results revealed that [1-^14^C]-NAE 12:0 was depleted by seedlings from the culture media but a significant increase in the amount of [1-^14^C]-NAE 12:0 removed from the media was observed when MDPD was included in the assays (Fig. 5b). This difference was most apparent midway through the time course at day 5 and 6 after stratification.

### MDPD rescues *AtFAAH* knockouts from the growth inhibitory effects of NAE 12:0

**Figure 6:**
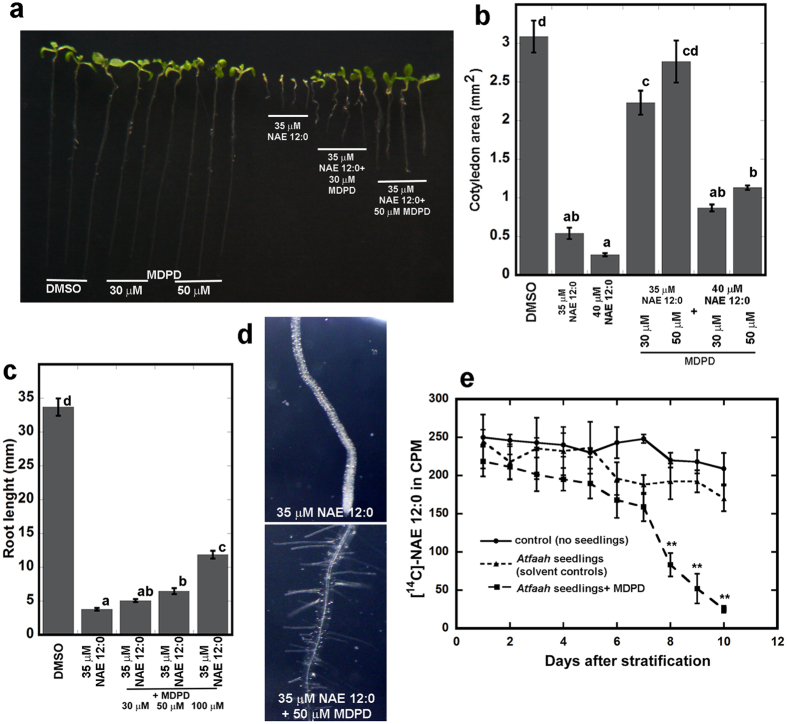
Effects of MDPD on NAE 12:0-induced seedling growth inhibition and *in vivo* [1-^14^C]-NAE 12:0 depletion assays in *Atfaah*. *Atfaah* seedlings were grown for 10 days (**a**) Representative images of 7-d old *Atfaah* seedlings on 35 μM NAE 12:0 alone or with 30 μM or 50 μM MDPD. Note that MDPD dampens the growth inhibitory effects of NAE 12:0 on *Atfaah*. (**b**) Primary root length on 35 μM NAE 12:0 alone or in combination with 30 μM, 50 μM or 100 μM MDPD. (**c**) Cotyledon area of *Atfaah* seedlings on media supplemented with 35 μM or 40 μM NAE 12:0 alone or in combination with 30 μM or 50 μM MDPD. Error bars represent the standard error of the means (*n* ≥ 16). Means with different letters are significantly different (P < 0.005; Tukey’s test). (**d**) Like in wild type, MDPD reversed the NAE 12:0-induced inhibition of root hair initiation in *Atfaah* seedlings. (**e**) The rate of depletion of radiolabeled NAE 12:0 is similar between negative controls (no seedlings) and media with *Atfaah* seedlings. With MDPD in the medium, radiolabeled NAE 12:0 is depleted after 7-8 days. Assays were performed with 6 *Atfaah* seedlings and 35 μM of radiolabeled NAE12:0 and 50 μM of MDPD, or an equal volume of DMSO, in 1.5 ml of MS liquid media. Student’s t-test (**indicates statistically significant differences, p < 0.05).

Our biochemical assays point to AtFAAH as a molecular target of MDPD. If AtFAAH is the only target of MDPD, it might be expected that the growth of *AtFAAH* knockouts (*Atfaah*)^17^ on NAE 12:0-containing media would not be enhanced by MDPD. To test this hypothesis, we conducted growth assays of *Atfaah* on NAE 12:0 and MDPD similar to those conducted on wild-type seedlings. Consistent with our previous results *Atfaah* seedlings were hypersensitive to the growth inhibitory effects of NAE 12:0^17^. Surprisingly, MDPD was still capable of attenuating the NAE 12:0-induced growth inhibitory effects on *Atfaah* seedlings and root hairs (Fig. 6a–d). These results with *Atfaah* indicated that MDPD likely affects other targets *in vivo* that might be involved in metabolizing NAE. Indeed, when we followed the depletion of [1-^14^C]-NAE 12:0 in liquid cultures, *Atfaah* seedlings alone were not able to deplete [1-^14^C]-NAE 12:0 efficiently from the liquid medium. However, when MDPD was included in the liquid medium, we observed a significant depletion of [1-^14^C]-NAE 12:0, but not until 8, 9, and 10 days after stratification (Fig. 6e). Hence, it appears that in *Atfaah*, MDPD is able to promote alternative means for seedlings to tolerate NAE 12:0, perhaps via the enhancement of other endogenous hydrolases.

### Structural analogs of MDPD do not enhance AtFAAH enzymatic activity

**Figure 7:**
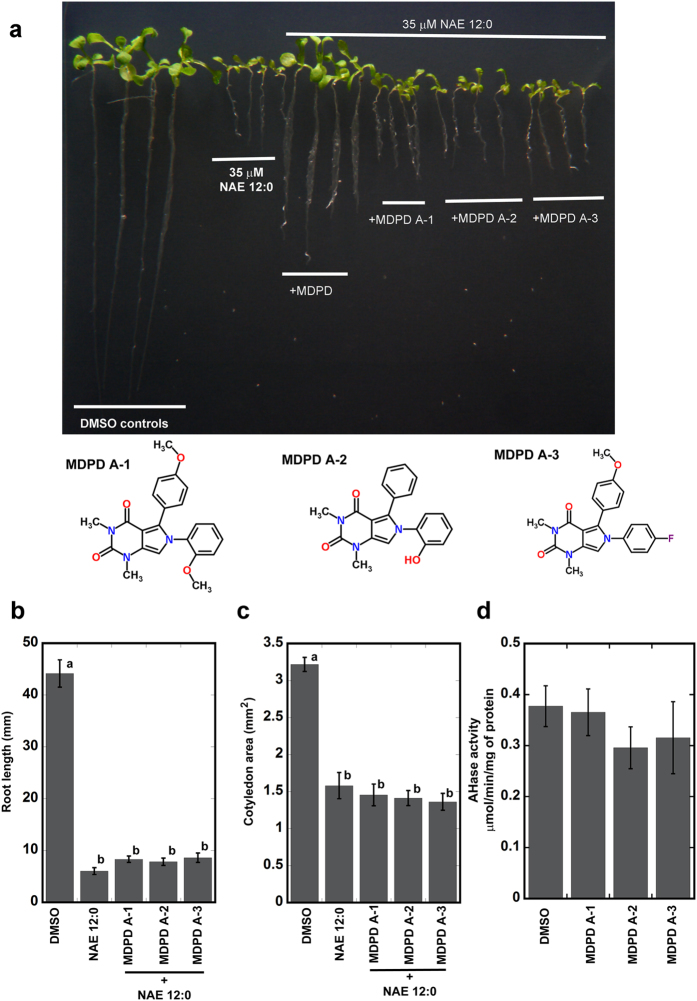
Effects of MDPD analogs on NAE 12:0 induced seedling growth inhibition. (**a**) Representative images of seedlings with the different treatments and the corresponding MDPD analogs they were treated with. Wild-type seedlings grown for 10 days on MS medium supplemented with 35 μM NAE 12:0 alone or in combination with 50 μM of the MDPD analogs. The structure of the MDPD analogs are shown below and are as follows: 6-(2-methoxyphenyl)-5-(4-methoxyphenyl)-1,3-dimethyl-1H-pyrrolo[3,4-d]pyrimidine-2,4(3 H,6 H)-dione (or **MDPD A-1**); MDPD A-2*** = ***6-(2-hydroxyphenyl)-1,3-dimethyl-5-phenyl-1H-pyrrolo[3,4-d]pyrimidine-2,4(3 H,6 H)-dione (or **MDPD A-2**), and 6-(4-fluorophenyl)-5-(4-methoxyphenyl)-1,3-dimethyl-1H-pyrrolo[3,4-d]pyrimidine-2,4(3 H,6 H)-dione (or **MDPD A-3**) NAE 12:0-induced root length (**b**) and cotyledon area (**c**) inhibition is not dampened by the analogs. Error bars represent the standard error of the means (*n* ≥ 120). (**d**) Amidohydrolase activity (AHase) with MDPD analogs and [1-^14^C]-NAE 12:0. Data points represent means ± S.D. of triplicate assays.

We investigated whether the antagonistic effects of MDPD on NAE 12:0 could be mirrored by structural analogs of MDPD. The molecules 6-(2-methoxyphenyl)-5-(4-methoxyphenyl)-1,3-dimethyl-1H-pyrrolo[3,4-d]pyrimidine-2,4(3 H,6 H)-dione (or MDPD A-1), 6-(2-hydroxyphenyl)-1,3-dimethyl-5-phenyl-1H-pyrrolo[3,4-d]pyrimidine-2,4(3 H,6 H)-dione (or MDPD A-2) and 6-(4-fluorophenyl)-5-(4-methoxyphenyl)-1,3-dimethyl-1H-pyrrolo[3,4-d]pyrimidine-2,4(3 H,6 H)-dione (or MDPD A-3) were about 95% similar to MDPD with regard to their 2-dimensional structures. These chemicals differed from MDPD only in the functional group substitutions on their phenyl rings (Fig. 7a). It was found that unlike MDPD, MDPD A-1, MDPD A-2 and MDPD A-3 did not relieve the growth inhibitory effects of NAE 12:0 on seedling growth (Fig. 7a–c). Furthermore, amidohydrolase assays *in vitro* using recombinant AtFAAH did not show enhanced enzymatic activity in the presence of these three MDPD analogs (Fig. 7d).

## Discussion

A chemical genetics approach led to the discovery of the small molecule, MDPD, which antagonizes the growth inhibitory effects of NAE 12:0 on *Arabidopsis* seedling growth. The biochemical assays presented here revealed that AtFAAH, a plant enzyme known to hydrolyze NAEs^15^,^17^, is one target of MDPD. Whereas all of the synthetic small molecules known to affect mammalian FAAH are inhibitory^27^, our studies are unique in that MDPD functions by enhancing the enzymatic activity of AtFAAH. Although the exact mode of action of this small molecule on AtFAAH is not fully understood, it does not seem to be through modulation of the affinity of AtFAAH for NAEs, but rather it appears to be an enhancement of the rate of NAE hydrolysis by AtFAAH by a factor of 3-to-4 times. We did not observe an increase in activity for the recombinant rat-FAAH protein in the presence of MDPD indicating that this small molecule is a specific modulator of plant FAAH. Differences between mammalian and plant FAAH in regard to efficacy of chemical agents are not without precedent. For example, in mammals, URB597 is a highly selective and irreversible antagonist of FAAH activity^20^, but this chemical did not alter FAAH activity in plants^16^. Despite the highly conserved amidase domain of plant and animal FAAH proteins^16^,^28^, differences in their enzymatic properties have been reported. For instance, rat-FAAH is a dual action enzyme that can also synthesize NAEs in the presence of large amounts of ethanolamine and FFA^29^. NAE synthesis by plant FAAH, however, has yet to be demonstrated. The identification of chemicals such as MDPD that selectively modify AtFAAH provides a new probe to better understand how mammalian FAAH differs from plant FAAH in regard to biochemical function.

Three structural analogs of MDPD did not prevent NAE 12:0-induced seedling growth inhibition nor did they stimulate AtFAAH activity *in vitro*. This indicates that MDPD is a highly specific antagonist of NAE 12:0-mediated seedling growth defects, and its impact on AtFAAH activity is likely determined by the functional groups of its phenyl rings. Interestingly, simply substituting the methoxyl group with a hydroxyl group in one phenyl ring, having methoxyl group for each phenyl ring, or halogenation of one of the phenyl rings led to the loss of MDPD’s ability to enhance AtFAAH activity and alleviate NAE 12:0 seedling effects. For the future, it would be important to determine how such structural modifications lead to loss of MDPD efficacy.

The increased AtFAAH enzymatic efficiency in the presence of MDPD is a likely explanation, at least in part, for its attenuation of NAE 12:0-induced seedling growth inhibition. This is supported by the observation that wild-type seedlings grown in the presence of NAE 12:0 and MDPD partly mimic *AtFAAH* overexpressors treated with NAE 12:0^17^. However, the fact that MDPD can still relieve the inhibitory effect of NAE 12:0 on *Atfaah*, albeit over a longer time scale, suggests that this synthetic molecule has other, yet-to-be–determined, targets in plants that might be involved in hydrolyzing NAEs. In support of this notion is the observation that *Atfaah* seedlings were not able to deplete radiolabeled NAE 12:0 efficiently from the liquid medium except when MDPD was added. The *Arabidopsis* genome encodes other enzymes with the conserved amidase signature domain^28^. Thus far, only amidase 1 (AMI1) has been shown to hydrolyze NAE but its activity was more directed toward indole-3 acetamide and only exhibited low activity toward NAE^30^. Recently, alternative pathways for the metabolic fate of NAEs have been discovered including the malonylation of glucose-conjugated NAE 12:0^31^ and lipoxygenase-mediated oxidation of polyunsaturated NAEs^32^,^33^. It is not clear how these additional NAE metabolic pathways interact with FAAH-mediated NAE hydrolysis. It is possible that in addition to its impact on other NAE hydrolases^28^, MDPD could have non-specific effects on these other NAE metabolic pathways that enable *Atfaah* to partially tolerate the inhibitory effects of NAE 12:0. Identification of other molecular targets of MDPD will be a priority for future research. Nonetheless, the discovery of MDPD as an AtFAAH-stimulating molecule opens new opportunities for probing deeper into the role of AtFAAH and NAE signaling in higher plants. Whereas, mammalian FAAH inhibitors have led to unprecedented insights into the effects of elevated NAE signaling on the endocannabinoid system^34^, not much is known about how dampened NAE signaling might influence cell physiology. Dampening of the NAE signal in *Arabidopsis* has been achieved by *AtFAAH* overexpression leading to the discovery of previously unknown functions for AtFAAH in plant response to pathogens, seedling development, cross-talk with hormones and flowering^11^,^12^,^17^,^35^. Chemical agents that stimulate AtFAAH should provide a nice complement to transgenic approaches to better understand how manipulation of the endogenous NAE tone impacts plant physiological processes.

## Methods

### Chemicals

[1-^14^C]-Lauric acid was from Amersham Biosciences, [1-^14^C]-palmitic acid was purchased from New England Nuclear (Boston, MA), and [1-^14^C]-arachidonic acid was purchased from PerkinElmer Life Sciences. Ethanolamine, anandamide, isopropyl-β-D-thiogalactopyranoside (IPTG), Triton-X100 were from Sigma Chemical Company (St. Louis, MO). N-dodecyl-β-D-maltoside (DDM) was from Calbiochem (LA Jolla, CA). Silica Gel G (60 A)-coated glass plates for thin-layer chromatography (10 cm × 20 cm or 20 cm × 20 cm, 0.25 mm thickness) were from Whatman (Clifton, NJ). Different species of *N*-[1-^14^C]-acylethanolamines (and non-radiolabeled NAEs) were synthesized from ethanolamine and corresponding [1-^14^C]-fatty acids (and non-radiolabeled FFAs) by first producing the fatty acid chloride^36^ and purified by TLC as described elsewhere^37^. The small molecule: 6-(2-methoxyphenyl)-1,3-dimethyl-5-phenyl-1H-pyrrolo[3,4-d]pyrimidine-2,4(3 H,6 H)-dione (MDPD), was purchased from Chembridge (San Diego, CA) and verified by Nuclear Magnetic Resonance (NMR) spectroscopy to be > 90% pure (Figure S1).

### Chemical library screen

A library containing 10,000 small organic molecules from Chembridge, San Diego was screened. Molecules were obtained in a 96-well-plate format at a 1 mM concentration. For the screen 100 μl of liquid 0.5 X Murashige and Skoog (MS) salt nutrient media supplemented with 1% sucrose and 50 μM NAE 12:0 was added with a multichannel pipette to 96-well plates. Approximately 4–5 wild type seeds were added to all the wells and chemicals from the library were then added to each of the columns in the 96-well plates at a 100 μM concentration with a multichannel pipette. The first and last columns of the plates were used as controls and no molecules were added to these wells. Each 96-well plates therefore contained 1 column of MS only media and 1 column of NAE 12:0 only media for comparison with seedlings treated with the small molecules. Plates were incubated for 6 days in a growth room with 16-h-light/8-h-dark cycle (60 *μ*mol.m^−2^.s^−1^) at 20 to 22 °C. Chemicals that dampened the inhibitory effects of NAE12:0 on wild-type seedling development were selected for more stringent rescreening.

### Seedling phenotypic assays

Wild-type Columbia-0 seeds were surface sterilized for 10 min in 30% bleach and rinsed four times with sterile deionized water. For seedling growth assays, seeds were plated on solid 0.5 X MS salts nutrient media supplemented with 1% sucrose alone or with 30, 35 or 40 μM NAE 12:0 and 30 μM, 50 μM or 100 μM MDPD. Plates were wrapped with parafilm, stratified for 48 h in dark at 4 °C and then incubated vertically in a growth room with 16-h-light/8-h-dark cycle (6 0 *μ*mol.m^−2^.s^−1^) at 20 to 22 °C. Images for primary root length and root hair elongation were taken at 10 d and 6 d, respectively. Primary root and root hair length, and cotyledon area were measured using ImageJ. For growth in liquid media, seedlings were grown in liquid MS media supplemented with 1% sucrose, and with different concentrations of NAE 12:0. Seedlings were grown for 3 days in the growth conditions described above then treated with 30 μM MDPD and allowed to grow for another 3 days. For MDPD pretreatment assays, seeds were grown in 30 μM MDPD for 3 days then transferred to media with 40 μM NAE 12:0 for an additional 3 days.

### Plasmid construction

The plasmid *AtFAAH-pTrcHis2 (At5g64440*) used for expression of AtFAAH protein for *in vitro* assays was described in Shrestha *et al*.^15^. The Rat-FAAH-pTrcHis2 (NP_077046) construct was a gift of Dr. Benjamin Cravatt^26^. The constructs were introduced by heat shock into chemically competent *E. coli* TOP10 cells as host.

### Protein expression and solubilization for enzymatic assays

*E. coli* cells were grown in 250 ml of Luria broth (LB) medium with 100 μg.ml^−1^ of filtered ampicillin to an A_600_ of 0.6, and induced with 1 mM IPTG for 4 hours at 37 °C. Each culture was centrifuged at 5000 rpm for 10 minutes at 4 °C. The pelleted cells expressing Rat-FAAH or AtFAAH were resuspended in 10 ml of lysis buffer A (50 mM Tris-HCl, 100 mM NaCl, 1% TritonX-100, pH8)). After incubation on ice for 30 min, resuspended cells were sonicated on ice with ten, 30-sec burst at 50% intensity with 30-sec cooling (ice) period between bursts. Each crude lysate was centrifuged at 13,000× *g* for 20 min at 4 °C in a Sorvall RC 5C model ultracentrifuge (Sorvall SS-34 rotor). The supernatant was applied to a QiQexpress NI-NTA Fast Start (Qiagen) column and the proteins were purified as according to the manufacturer’s instructions. The purified fractions (2 ml) were concentrated, and imidazole was removed with buffer B (50 mM Bis-Tris propane, pH9, 0.2 mM dodecyl maltoside (DDM)) by filtration-centrifugation using Centricon YM-30 (Millipore, Bedford, MA) devices (Triton X-100, instead of DDM, was substituted for the recovery of active rat enzyme). The protein concentration was estimated by Bradford assays (Sigma St. Louis) against a BSA standard curve, and the purity of the recombinant, epitope-tagged proteins were evaluated by SDS-PAGE gel and Western blotting.

### Plant protein extraction

Wild-type seeds were germinated in 75 ml of liquid culture for 10 days under short day conditions for protein extraction. After 10 days in liquid culture, seedlings were collected and rinsed with milliQ water and then blotted dry. Using a mortar and pestle, 500 mg of seedlings were ground in liquid nitrogen and placed in 2 ml of plant protein solubilization solution (0.1 M potassium phosphate buffer, pH7.2, 400 mM sucrose, 10 mM KCl, 1 mM EDTA, 1 mM EGTA, 1 mM MgCl_2_) with 0.2 mM of DDM. The crude extract was vortexed for 1 min then incubated at 4 °C for 30 min while shaking. The crude extract was centrifuged at 600× *g* at 4 °C for 10 min (Beckman CPR model, GH 3.7 rotor). The supernatant, containing the total solubilized proteins, was stored at 4 °C until used for enzyme assays.

### SDS-PAGE and Western Blotting

Rat or AtFAAH protein aliquots were separated by SDS-PAGE (10% resolving gels) according to Shrestha *et al*.^15^. The proteins were visualized in gels by Coomassie-blue staining, or proteins were electrophoreticaly transferred to polyvinylidene fluoride (PVDF) membranes (0.2 μm, Bio-Rad, Hercules, CA) as previously described^16^. The recombinant proteins expressing the HIS tag at the C-terminus were detected by chemiluminescence using a 1-to-2000 dilution of mouse monoclonal anti-HIS antibodies (ABGENT San Diego, CA) and a solution of 1-to-4000 dilution of goat anti-mouse IgG conjugated to a peroxidase (Bio-Rad).

### *In vitro* assays of purified FAAH proteins and whole plant protein extracts

The NAE amidohydrolase assays were conducted as previously described Shrestha *et al*.^15^ with a few modifications. The reactions were conducted for 30 min at 30 °C or 37 °C (AtFAAH and Rat-FAAH, respectively), in 150 μl of buffer B containing different concentrations of radiolabelled NAEs, MDPD and different concentrations of purified protein. Reactions typically were initiated by the addition of enzyme (0.5-2 micrograms). Enzyme reactions were terminated by the addition of hot isopropanol (70 °C). The lipids were extracted and the distribution of the radioactivity in lipids was evaluated by radiometric scanning of TLC plates as previously described^16^. Enzymatic assays from crude plant protein extracts were conducted at 30 °C for 2 hours in buffer B with 300 μM of MDPD and 200 μM of radiolabeled NAE 12:0. The reactions were started by adding 5 mg of total protein and were stopped by adding hot isopropanol and the lipid content were extracted and analyzed as above.

### *In vivo* NAE depletion assays

Using a 12 well plate, 1.5 ml of regular MS media supplemented with 35 μM of non-radiolabeled NAE12:0 were added first to saturate reactive sites with NAE 12:0. After a few minutes, the contents of each well were discarded and six seeds, were grown in 1.5 ml of regular MS media supplemented with 35 μM of [1-^14^C]-NAE12:0 and 50 μM of MDPD. Seeds were stratified in the dark at 4 °C for 3 d. Seedlings were grown under short day conditions (9 hours) with agitation (35 rpm). During the time course, radioactivity was measured in 20 μl aliquots from each well by liquid scintillation counting (Beckman LS6000IC) in 3 mL of ScintiSafe Plus^TM^ 50% (Fisher).

## Additional Information

**How to cite this article**: Khan, B. R. *et al*. A chemical genetic screen uncovers a small molecule enhancer of the *N*-acylethanolamine degrading enzyme, fatty acid amide hydrolase, in Arabidopsis. *Sci. Rep.* **7**, 41121; doi: 10.1038/srep41121 (2017).

**Publisher's note:** Springer Nature remains neutral with regard to jurisdictional claims in published maps and institutional affiliations.

## Supplementary Material

Supplementary Figures
