# Supplementary material for: A chemical genetic screen uncovers a small molecule enhancer of the N-acylethanolamine degrading enzyme, fatty acid amide hydrolase, in Arabidopsis
Source: Sci Rep. 2017 Jan 23;7:41121. doi: 10.1038/srep41121 (PMC5253734; doi:10.1038/srep41121)
Supplement: Supplementary Figures [file srep41121-s1.pdf]

Supplemental Information

A chemical genetic screen uncovers a small molecule enhancer of the *N*-acylethanolamine degrading enzyme, fatty acid amide hydrolase, in Arabidopsis

Authors: Bibi Rafeiza Khan, Lionel Faure, Kent D. Chapman and Elison B. Blancaflor

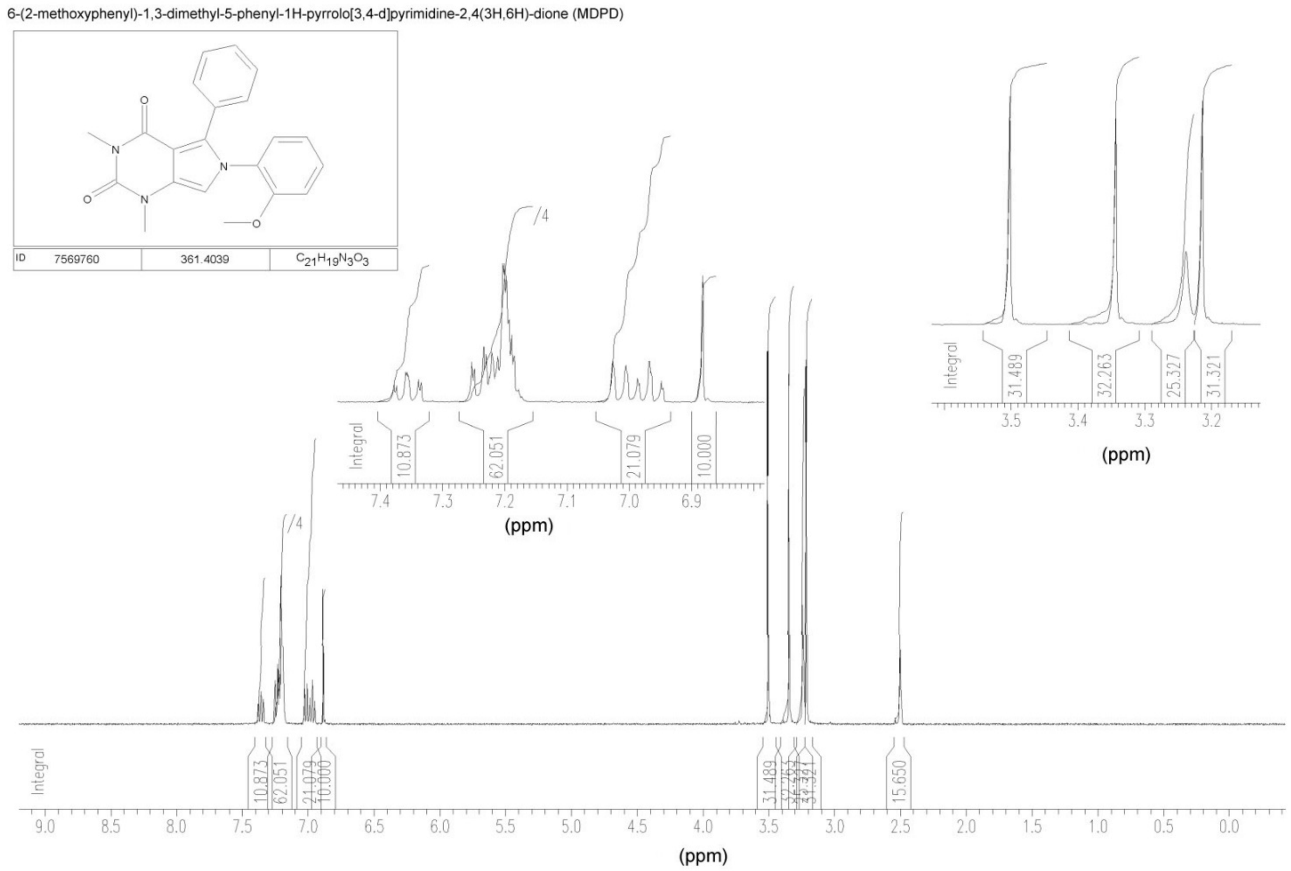

Figure S1. Analytical information demonstrating the chemical identity and purity of MDPD by Nuclear Magnetic Resonance (NMR) spectroscopy. NMR analyses indicates >90% purity of the MDPD compound used in this study. NMR spectra courtesy of ChemBridge (San Diego, CA).

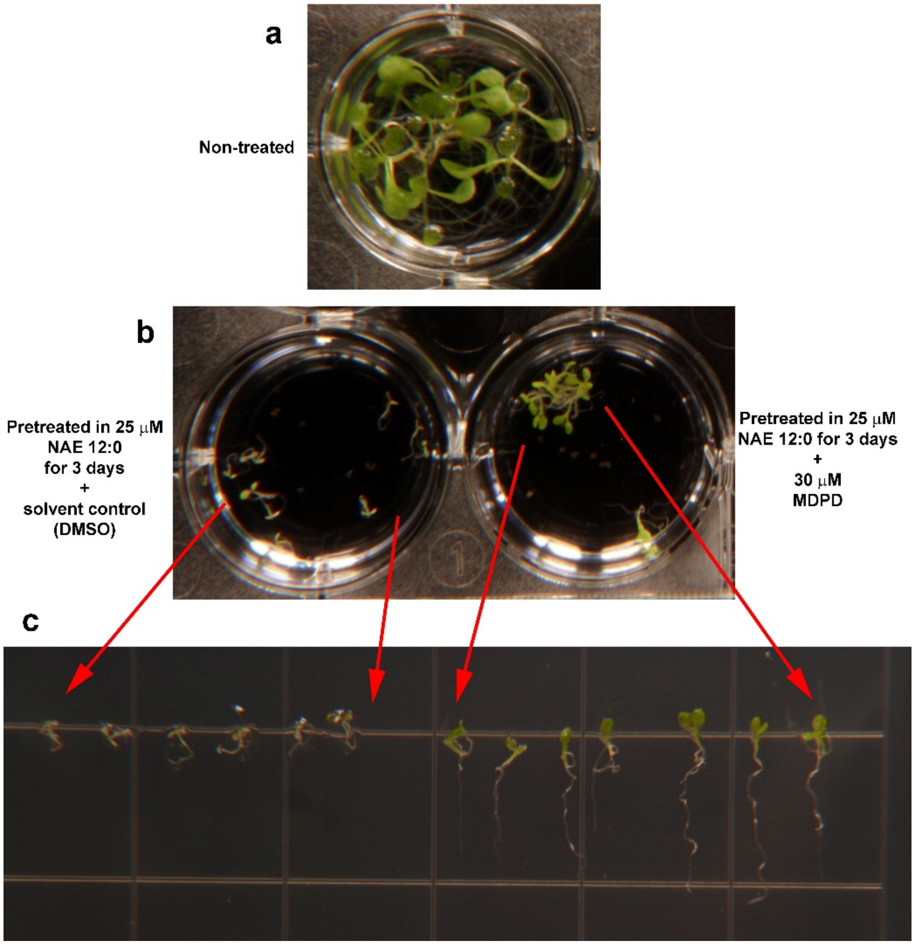

Figure S2. Effects of MDPD on wild-type *Arabidopsis* seedlings pretreated with NAE for 3 days. Wild type seedlings grown in liquid media for a total of 6 days. (a) No treatment; (b) Seedlings grown in 25 μM NAE 12:0 for 3 d then supplemented with 30 μM MDPD; (c) High magnification image of seedlings from (b) after transfer to solid media.

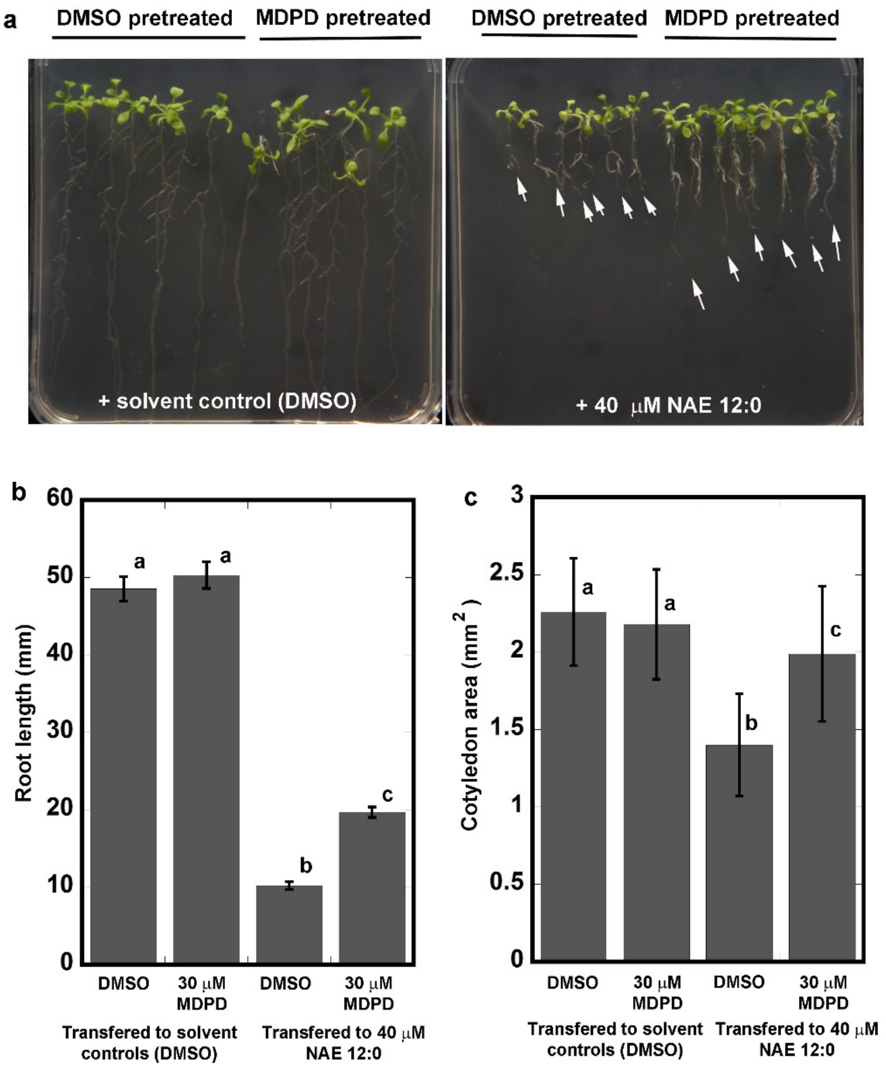

Figure S3. Effects of NAE 12:0 on seedling development after pretreatment with MDPD. Wild-type seeds were grown in 0.5% MS only media or media supplemented with 30 µM MDPD for 3 days. Seedlings were then transferred to media with or without 40 µM NAE 12:0. (a) Representative images of seedlings 4 days after transfer to NAE 12:0 supplemented plates. Quantification of primary root length (b) and cotyledon area (c). Note that MDPD pretreatment dampens primary root and cotyledon area growth inhibition resulting from transfer to NAE 12:0 Error bars represent the standard error of the means ($n \geq 10$). Means with different letters are significantly different ($P < 0.005$; Tukey's test).
